# Supplementary material for: A set of multi-entry identification keys to African frugivorous flies (Diptera, Tephritidae)
Source: Zookeys. 2014 Jul 24;(428):97–108. doi: 10.3897/zookeys.428.7366 (PMC4143993; doi:10.3897/zookeys.428.7366)
Supplement: Supplementary material 6 — Key to Ceratitis [file zookeys-428-097-s006.zip › SF6_ZooKeys_key to Ceratitis/key/SF6_ZooKeys_key to Ceratitis/Media/Html/Ceratitis perisae.htm]

***Ceratitis*** *(**Ceratalaspis**)
**perisae*** **De Meyer & Copeland**

*Ceratitis (Ceratalaspis) perisae* De Meyer
& Copeland, 2005 : 1289.

Body
length. 4.60 (4.50-4.70) mm; wing length. 4.83 (4.75-4.90) mm.

Female

Head.
Antenna pale yellow. First flagellomere darker yellow; 3 times as long as
pedicel. Arista with short rays, longest dorsal rays about as long as basal
width of arista. Frons pale yellow; with short scattered yellow setulae; two
frontal setae. Face white. Genal setulae darkish, poorly developed; genal seta
reddish.

Thorax.
Postpronotal lobe yellowish white; without spot. Scutum, ground colour
yellow-orange, with distinct brown to black markings but these restricted to
intra-alar spot and spot linking dorsocentral and acrostichal prescutellar setae,
sutural spot brownish, other dark markings pale brownish; prescutellar markings
white, separate; with yellow microtrichosity and pilosity. Scapular setae
black. Scutellum white, basally without black spots but with yellow-brown
marking, apically with three separate black spots, extending to basal third.
Subscutellum yellow with three separate black spots, touching along dorsal
margin. Anepisternum yellow; one black anepisternal seta, setulae pale.

Legs.
Yellow; setation yellow to orange except last tarsal segments black. Fore femur
with ventral setae yellow, posteriorly with longer pilosity.

Wing.
Bands mainly yellowish with some brown patches. Anterior apical and discal
bands not separate; subapical band joined to discal band; posterior apical band
joined to anterior apical band; crossvein R-M at proximal two-fifths of discal
medial cell.

Abdomen.
Yellow to orange; pilosity mainly black. Tergites 2 and 4 with pale grey band
occupying posterior third of tergite. Tergite 5 with longer black setulae along
posterior margin. Oviscape shorter than� preabdomen. Aculeus 6-7 times longer
than wide, apical part with three large rounded indentations, tip with small
depression.

Male

As
female except for the following characters. First flagellomere pale yellow. Mesonotal
pattern with sutural spot also pale brownish, male paratype of Ngaia Forest
with very weak mediolongitudinal line postsuturally. Scutellum basally with
yellow marking. Subscutellum with black spots separate. Wing, crossvein R-M at
proximal third of discal medial cell.

(Description
after De Meyer & Copeland, 2005)
